# Effects of mutations in *SGS1 *and in genes functionally related to *SGS1 *on inverted repeat-stimulated spontaneous unequal sister-chromatid exchange in yeast

**DOI:** 10.1186/1471-2199-8-120

**Published:** 2007-12-31

**Authors:** Dilip K Nag, Steffany J Cavallo

**Affiliations:** 1 Division of Molecular Medicine, Wadsworth Center, Center for Medical Sciences, 150 New Scotland Avenue, Albany, NY 12208, USA; 2 Department of Biomedical Sciences, School of Public Health, State University of New York, Albany, NY 12201, USA

## Abstract

**Background:**

The presence of inverted repeats (IRs) in DNA poses an obstacle to the normal progression of the DNA replication machinery, because these sequences can form secondary structures ahead of the replication fork. A failure to process and to restart the stalled replication machinery can lead to the loss of genome integrity. Consistently, IRs have been found to be associated with a high level of genome rearrangements, including deletions, translocations, inversions, and a high rate of sister-chromatid exchange (SCE). The RecQ helicase Sgs1, in *Saccharomyces cerevisiae*, is believed to act on stalled replication forks. To determine the role of Sgs1 when the replication machinery stalls at the secondary structure, we measured the rates of IR-associated and non-IR-associated spontaneous unequal SCE events in the *sgs1 *mutant, and in strains bearing mutations in genes that are functionally related to *SGS1*.

**Results:**

The rate of SCE in *sgs1 *cells for both IR and non-IR-containing substrates was higher than the rate in the wild-type background. The *srs2 *and *mus81 *mutations had modest effects, compared to *sgs1*. The *exo1 *mutation increased SCE rates for both substrates. The *sgs1 exo1 *double mutant exhibited synergistic effects on spontaneous SCE. The IR-associated SCE events in *sgs1 *cells were partially *MSH2*-dependent.

**Conclusions:**

These results suggest that Sgs1 suppresses spontaneous unequal SCE, and *SGS1 *and *EXO1 *regulate spontaneous SCE by independent mechanisms. The mismatch repair proteins, in contradistinction to their roles in mutation avoidance, promote secondary structure-associated genetic instability.

## Background

During DNA replication, the extension of daughter strands is continuously impaired by a number of factors, such as proteins bound to the template, endogenously or exogenously induced DNA damage, and the presence of DNA secondary structures. If the replication fork stalls, and if the stalled fork is not processed to restore fork progression, disassembly of the replication complex can ensue. The stalled forks can also break, generating a double-strand break (DSB). Additionally, the presence of a DNA lesion, such as a single-strand nick in the template strand, can lead to a DSB. Consequently, a failure to repair the replication-associated lesions, and to then restart the stalled fork, will lead to chromosome loss or impairment of the integrity of the genome. Maintenance of the stability of the genome is critical for normal cell growth and cell viability.

To avoid genetic instability, cells have evolved a variety of mechanisms to rescue the stalled fork; extensive studies in both prokaryotes and eukaryotes suggest that homologous recombination plays a critical role in repair of the replication-associated DNA lesions, and in allowing the replication to continue [1-3]. For example, DSBs arising as a result of replication defects can be repaired by homologous recombination, using the sister chromatid as a template. Similarly, a replication fork stalled due to the presence of a replication block can be reinitiated by a template-switching mechanism, before the replication block is removed. However, unscheduled recombination can be detrimental, leading to a higher rate of genetic instability, as observed in the cancer-prone Bloom, Werner, and Rothmund-Thomson syndromes, respectively due to mutations in the *BLM*, *WRN*, and *RECQL4 *genes [4]. These three genes belong to a highly conserved family of RecQ DNA helicases, originally described in *Escherichia coli *as a component of the RecF recombination pathway [4,5].

BLM cells show a high rate of sister-chromatid exchange (SCE), and the sensitivity of both BLM and WRN cells to S-phase-specific inhibitors (*e.g*., camptothecin) suggests that these genes function during DNA replication [4]. In addition, there is mounting evidence in yeast suggesting that replication does not proceed normally in the absence of RecQ helicases. Cells lacking the RecQ homolog Sgs1 in *Saccharomyces cerevisiae *exhibit an increased sensitivity to DNA-damaging agents (*e.g*., ultraviolet light, hydroxyurea, and methyl-methane sulphonate); an increased level of recombination between homologous sequences and between modestly divergent DNA sequences; gross chromosomal rearrangements; unequal SCE; and mitotic chromosome non-disjunction [6-12]. The Sgs1 protein closely associates with the replication fork and is thought to stabilize and restart the stalled fork [13-15]. *In vitro *studies have indicated that Sgs1, like its human counterpart, is a 3'-5' DNA helicase that can disrupt a variety of DNA structures, including cruciform structures that resemble the Holliday junction intermediate of the recombination process, suggesting its possible role in homologous recombination [16]. Sgs1 physically interacts with type I topoisomerase I (Top3), and both genetic and biochemical studies indicate that the Sgs1/Top3 complex acts on Holliday junctions to suppress crossover outcomes [17-20].

Several synthetic lethal screens have been employed to identify the genes that are functionally related to Sgs1 [21-27]. The *sgs1 *mutation is synthetically lethal with a mutation in the *SRS2 *gene, which encodes another 3'-5' DNA helicase [26]. Cells lacking Sgs1 and Srs2 are extremely sick, and the growth defect is suppressed by a mutation in any of the *RAD51, RAD52, RAD55*, and *RAD57 *genes involved in early stages of homologous recombination [21,24,27,28]. Since the Sgs1 and Srs2 proteins function during DNA replication [4,29], it has been proposed that Sgs1 and Srs2 in wild-type cells regulate the accumulation of toxic recombination intermediates, during DNA replication. *In vitro *studies have shown that Srs2 possesses an anti-recombination activity; it displaces Rad51, a strand-annealing protein, from DNA filaments [30,31], which is in agreement with Srs2's *in vivo *recombination-inhibiting activity [32].

The *sgs1 *mutation is also synthetically lethal with *mus81*, but a *rad51 *mutation suppresses the lethal effect of the double mutation [22,24], suggesting that Sgs1 and Mus81 function in separate pathways. Mus81 acts in a complex with Mms4; the heterodimeric protein has been shown to cleave branched DNAs and has been implicated in DNA repair; it also functions during sporulation [33-35]. Results of several genetic studies have led to the proposal that DNA structures formed during replication are acted upon by recombination proteins, forming intermediates that are toxic unless processed further. Sgs1/Top3 and Mus81 are needed to process these intermediates, whereas Srs2 limits the numbers of such intermediates, by disrupting Rad51 filaments.

In eukaryotes, SCE occurs spontaneously, probably representing recombination events during replication. The factors that impair the normal progression of the replication fork are likely to increase the rate of spontaneous SCE. One of the factors that compromises the normal progression of the replication fork is the presence of inverted repeats (IRs) that can form secondary structures in single-stranded DNA, by intra-strand base pairing between complementary sequences. Consistently, IRs have been found to be associated with gross chromosomal rearrangements [36,37]. Previously, we constructed a recombination substrate to study the effect of IRs on unequal SCE in haploid *S. cerevisiae *[38]. The presence of the repeated sequences increases spontaneous unequal SCE by about 10-fold [38,39]. While non-IR-mediated SCE events are independent of DSB-repair genes, IR-stimulated SCE events depend on DSB repair genes, suggesting that IR-associated SCEs occur by DSB repair [38].

During DNA replication, the lagging strand is expected to contain a higher level of single-stranded regions than does the leading strand, due to the discontinuous nature of DNA synthesis. The single-stranded regions facilitate the formation of a secondary structure at an IR. The secondary structures are also substrates for structure-specific nucleases *in vivo*. Cleavage of the secondary structure at the stalled fork will lead to the formation of a DSB that can be repaired by either gene conversion or break-induced replication [1], using the sister chromatid as a template. In the present study, we analyzed IR-stimulated unequal SCE in cells lacking Sgs1 and/or functionally related enzymes that are believed to function at the stalled replication forks. Our results showed that the *sgs1 *mutation increases unequal SCE for both IR-containing and non-IR-containing substrates. However, IR-stimulated SCE events in the *sgs1 *background are significantly reduced when defects in the mismatch repair (MMR) gene *MSH2 *are also present. Additionally, we showed that *SGS1 *and *EXO1 *regulate SCE in two distinct pathways.

## Results

### The *sgs1 *mutation increases the rates of spontaneous unequal SCE of both the non-IR-containing and IR-containing substrates, whereas the *srs2 *and *mus81 *mutations produce much smaller effects

Recombination between sister chromatids occurs spontaneously, and the majority of these recombination events are likely to occur during DNA replication. Since a secondary structure is likely to act as an obstacle to the progression of the replication fork, DNA sequence elements that have the potential to form secondary structures have been shown to compromise DNA replication both *in vivo *and *in vitro *[40-44]. A replication block is expected to increase the level of SCE. Accordingly, both IRs and trinucleotide repeats that can form hairpin or cruciform structures have been shown to increase the rate of spontaneous unequal SCE [38]. Since Sgs1 has been proposed to play a role in stabilizing and restarting a stalled fork [13-15], we sought to determine the effect of the *sgs1 *mutation on IR-stimulated SCE.

**Figure 1:**
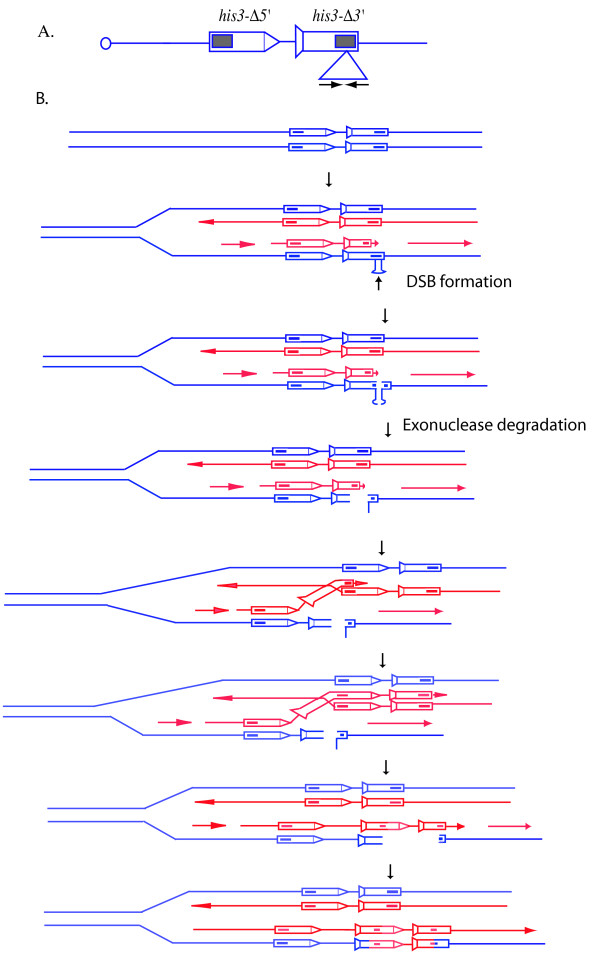
Unequal SCE assay. A. The *his3 *substrate for measurement of unequal SCE. The *his3-Δ3' *construct is marked with a tail, and the *his3-Δ5' *construct is marked with an arrowhead. The shaded region indicates the regions shared by the two deletion constructs. Expanded region under the linear map represents the palindromic insertion. B. DSB repair by gene conversion. A DSB is formed when the replication fork has stalled at the secondary structure. Although a secondary structure can form on both the lagging and the leading strand, the discontinuous nature of DNA synthesis is likely to facilitate formation of greater amounts of secondary structures on the lagging strand than on the leading strand. Shown here is the repair of a DSB formed on the lagging strand via unequal SCE, using the sister chromatid as a template. The unequal SCE events generate a wild-type *HIS3 *gene. DSBs can also be repaired by equal SCE. However, equal SCE will not give rise to a wild-type *HIS3 *gene. DSBs are likely to form via the endonuclease activity of a structure-specific nuclease.

Equal SCE is difficult to follow due to the identical nature of the sister chromatids. We measured unequal SCE, employing a *his3 *sister-chromatid recombination substrate (*his3-SCS*) that consists of two tandem copies of truncated *his3 *fragments: one fragment lacks the 5' end of the gene (*his3-Δ5'*), and the other lacks the 3' end (*his3-Δ3'*). The two deletion fragments share a 508-bp sequence homology (Fig. 1). An unequal recombination between the two fragments will generate a wild-type *HIS3 *gene. To determine the effect of the presence of an IR on SCE, we introduced a 140-bp IR in the *his3-Δ*3' construct within the region shared by the two deletion constructs, to generate the *his3-SCS*_*pal*140 _substrate [38]. A control substrate (*his3-SCS*_*control*_) was generated by insertion of a 117-bp non-repeated sequence within the region of homology in the *his3-Δ*3' construct [38]. Previously, we showed that the presence of an IR stimulates unequal SCE by about 10-fold over the rate for the control substrate [38,39]. It has also been shown that the IR-stimulated SCE events occur via DSB repair (Fig. 1) [38].

We introduced the *sgs1 *mutation into haploid strains (Table 1) containing either the *his3-SCS*_*control *_or *his3-SCS*_*pal*140 _substrate, and we then determined the rates of spontaneous unequal SCE as described in Methods. The rate of spontaneous unequal SCE in *sgs1 *cells was increased nearly 14-fold for the control substrate, and 11-fold for the IR-containing substrate over the rate for the wild-type strain (Table 2). Since the Sgs1 and Srs2 helicases are functionally related, and since both play roles in regulating mitotic crossover events, we introduced the *srs2 *mutation in our strains, and then measured the rates of SCE. Unlike the *sgs1-*mutant cells, *srs2 *cells exhibited a modest increase in SCE; the rate of SCE for *his3-SCS*_*control *_and *his3-SCS*_*pal*140 _was increased 3.3- and 2-fold, respectively, compared to the corresponding wild-type rates (Table 2).

**Table 1:** Yeast strains used in this study.

Strain	Genotype
AS13	*MAT***a ***leu2-Bst ura3-52 ade6*
DNY380	AS13 *lys2 arg4 his3Δ arg4::his3-SCS*_*control*_
ATY1	DNY380 *sgs1*
DNY438	DNY380 *srs2*
DNY446	DNY380 *mus81*
DNY443	DNY380 *sgs1 rad51*
DNY450	DNY380 *sgs1 exo1*
DNY452	DNY380 *sgs1 msh2*
DNY471	DNY380 *sgs1 rad52*
DNY393	AS13 *lys2 arg4 his3Δ arg4::his3-SCS*_*pal*140_
ATY2	DNY393 *sgs1*
DNY442	DNY393 *srs2*
DNY447	DNY393 *mus81*
DNY439	DNY393 *sgs1 rad51*
DNY451	DNY393 *sgs1 exo1*
DNY453	DNY393 *sgs1 msh2*
DNY472	DNY393 *sgs1 rad52*

**Table 2:** Rates of unequal SCE in various genetic backgrounds.

Genotype	SCE rate for *his3-SCS*_*control *_(× 10^6^)	Relative rate	SCE rate for *his3-SCS*_*pal*140 _(x10^6^)	Relative rate
^a^Wild type	0.72 ± 0.06	1.0	6.66 ± 0.44	1.0
*sgs1*	9.95 ± 1.50	13.8 ↑ (P < 0.0001)	71.3 ± 8.44	10.7 ↑ (P < 0.0001)
*srs2*	2.38 ± 0.64	3.30 ↑ (P < 0.0001)	13.4 ± 2.24	2.01↑ (P = 0.0001)
*mus81*	1.18 ± 0.20	1.64 ↑ (P = 0.0005)	8.64 ± 1.19	1.30 ↑ (P = 0.006)
^a^*exo1*	2.80 ± 0.67	3.88 ↑ (P < 0.0001)	26.9 ± 5.93	4.03 ↑ (P < 0.0001)
^b^*rad51*	1.36 ± 0.38	1.90 ↑ (P < 0.0001)	2.70 ± 0.20	0.40 ↓ (P < 0.0001)
^b^*rad52*	0.16 ± 0.02	0.22 ↓ (P < 0.0001)	0.58 ± 0.02	0.08 ↓ (P < 0.0001)
^a^*msh2*	1.87 ± 0.33	2.59 ↑ (P < 0.0001)	4.18 ± 0.46	0.62 ↓ (P < 0.0001)
*sgs1 rad51*	4.53 ± 1.07	6.29 ↑ (P < 0.0001)	13.7 ± 2.68	2.06 ↑ (P = 0.0002)
*sgs1 rad52*	1.32 ± 0.76	1.8 ↑ (P = 0.050)	0.75 ± 0.18	0.11 ↓ (P < 0.0001)
*sgs1 exo1*	21.8 ± 3.36	30.27 ↑ (P < 0.0001)	150 ± 13.0	22.52 ↑ (P < 0.0001)
*sgs1 msh2*	10.0 ± 2.66	13.9 ↑ (P < 0.0001)	35.3 ± 6.05	5.30 ↑ (P < 0.0001)

^a^Rates obtained from ref. 39.

^b^Rates obtained from ref. 38.

The *sgs1 *deletion is synthetically lethal with *mus81 *and *mms4 *[22,24], and mutations in the homologous recombination genes suppress the lethal effect of the double mutation [24]. Since Mus81 has been shown to possess a structure-specific endonuclease activity [33], this endoncuclease is therefore a candidate to generate DSBs at the secondary structure during DNA replication. To determine the role of Mus81 in IR-associated SCE, we determined the rates of unequal SCE in *mus81 *cells. Both the control and the IR-containing substrates exhibited a slight increase (1.6 and 1.3-fold, respectively) in SCE rates as compared to the rates in wild-type cells, suggesting that IR-stimulated SCE is not due to Mus81-generated DSBs at secondary structures during replication.

### Both *RAD51*-dependent and *RAD51*-independent recombination events are responsible for elevated SCE in *sgs1 *cells

The lethal phenotype of the *sgs1 srs2 *double mutant can be suppressed by a mutation in the *RAD51 *gene [21], which encodes the strand-exchange protein of the homologous recombination machinery [28]. This result further implies that Rad51 functions upstream of where Sgs1 acts. Therefore, one would expect that the simultaneous deletion of *RAD51 *and *SGS1 *will generate a SCE level that is similar to that of the *rad51 *single mutant. The rate of spontaneous SCE for *his3-SCS*_*control *_is similar in the wild type and in the *rad51 *background (38). The rate of SCE for *his3-SCS*_*pal*140 _is reduced in the *rad51 *background compared to wild-type cells, because IR-stimulated SCE events occur by DSB repair. We analyzed spontaneous unequal SCE in the *rad51 sgs1 *double mutant for both *his3-SCS*_*control *_and *his3-SCS*_*pal*140 _substrates. The rate of SCE for the *his3-SCS*_*control *_substrate in the double mutant was reduced to 50% of the *sgs1 *level, but it still remained 3.3 times higher than the rate in *rad51 *cells (Table 2)[38]. The *rad51 *mutation had a greater effect on IR-stimulated SCE than on non-IR-associated SCE; the rate of SCE was reduced to 19% of the *sgs1 *level, but remained 5-fold higher than the *rad51 *level. These results suggest that a proportion of the SCE events that occur in *sgs1 *cells, for both the control and the IR-containing substrates, are *RAD51*-independent.

Recombination in *S. cerevisiae *occurs in both *RAD51*-dependent and *RAD51*-independent pathways, but both pathways are dependent on *RAD52 *(28). We therefore sought to determine whether *RAD51*-independent events in the *sgs1 *background are *RAD52*-dependent. The rate of unequal SCE was reduced, for both substrates, in the *sgs1 rad52 *background. The rate of SCE for the IR-containing substrate, in the *sgs1 rad52 *background, was reduced nearly to the *rad52 *level, whereas the rate of SCE for the control substrate in the double mutant remained about 8-fold higher than the *rad52 *level (Table 2). These results suggest that most of the *RAD51*-independent IR-associated SCE events in the *sgs1 *background are *RAD52*-dependent, and also that some SCE events for the control substrate in *sgs1 *cells occur via a *RAD52*-independent pathway.

### IR-associated SCE events in the *sgs1 *background are *MSH2*-dependent

Msh2 is a key component of the MMR complex [45]. In *S. cerevisiae*, three *MSH *genes (*MSH2*, *MSH3*, and *MSH6*) are involved in MMR. Mismatch recognition is accomplished by Msh2-Msh3 and Msh2-Msh6 heterodimers. The Msh2-Msh6 complex shows strong selectivity for base-base and single insertion/deletion mismatches, while the Msh2-Msh3 complex preferentially recognizes small loops. Previously, we showed that IR-stimulated SCE events are reduced in *msh2 *and *msh3 *backgrounds; none of the other proteins involved in the MMR pathway is required for these events [39]. IR-mediated spontaneous SCE events are reduced 2-fold in the *msh2 *and *msh3 *backgrounds, while the rate of SCE for *his3-SCS*_*control *_is increased nearly 2.6-fold in *msh2 *cells. It is not known how *MSH2 *regulates the secondary structure-related SCE. Since IR-associated SCE events occur via DSB repair, Msh2 may act before or after the generation of DSBs. Sgs1 is known to interact with MMR proteins [46-48].

If the increased level of IR-associated SCE events in *sgs1 *cells occurs via the same mechanism as in wild-type cells, then these events should be Msh2-dependent. We measured the rates of SCE for both *his3-SCS*_*control *_and *his3-SCS*_*pal*140 _in a *sgs1 msh2 *double mutant. While the rate of SCE in the double mutant for the *his3-SCS*_*control *_substrate remained at the *sgs1 *(single mutant) level (P = 0.48), the level of SCE for the *his3-SCS*_*pal*140 _substrate was reduced by 50% (Table 2) in the double mutant as compared to the *sgs1 *single mutant (P < 0.0001), suggesting that half of the IR-mediated SCE events in *sgs1 *cells occur in an *MSH2*-dependent pathway, and that Msh2 acts upstream of Sgs1. These results also indicate that the increased levels of SCE events for *his3-SCS*_*control *_and *his3-SCS*_*pal*140 _in the *sgs1 *background occur by differing mechanisms, and that Sgs1 suppresses both *MSH2*-dependent and *MSH2*-independent IR-associated events in wild-type cells.

### *SGS1 *and *EXO1 *regulate spontaneous SCE events by independent pathways

Exo1, a 5'-3' exonuclease that acts preferentially on duplex DNA, has been implicated in MMR and in recombination, and it has also been shown to act on stalled replication forks [49,50]. Exo1 has also functional redundancy with flap endonuclease Rad27 for processing of the Okazaki fragments [51]. A failure to restart the stalled fork, or to process the Okazaki fragments, is expected to raise the level of SCE. Accordingly, it has been shown that the *exo1 *mutation increases spontaneous SCE for both control and IR-containing substrates by 4-fold over the rate in wild-type cells (Table 2, [39]). Sgs1 is believed to stabilize the stalled replication fork, and to act on the recombination intermediates that are generated due to single-stranded gaps formed during DNA replication [24]. It is possible that Sgs1 and Exo1 are epistatic. A synthetic interaction between *sgs1 *and *exo1 *that causes fitness defects has been reported [25]. The synthetic interaction further suggests a role of *exo1 *in repair and restart of the stalled replication forks. In our strain background, the *sgs1 exo1 *double mutant was viable; the double mutant grew only slightly more slowly than did the *sgs1 *single mutant. The synthetic interaction between *sgs1 *and *exo1 *may also be dependent on the strain background.

We monitored spontaneous SCE for both the *his3-SCS*_*control *_and *his3-SCS*_*pal*140 _substrates in the *sgs1 exo1 *double mutant. The rates of spontaneous unequal SCE for *his3-SCS*_*control *_in the double mutant was increased 30-fold over the wild-type level, whereas the level of SCE in the *sgs1 *single mutant was 14-fold higher than the wild-type level (Table 2). The rates of SCE for *his3-SCS*_*pal*140 _in the *sgs1 *single mutant and *sgs1 exo1 *double mutant were respectively, 11- and 23-fold increased over the rate observed in the wild-type background. These results indicate that Sgs1 and Exo1 regulate spontaneous SCE in two independent pathways.

## Discussion

Inverted repeats that have the potential to form secondary structures provide an excellent system in which to study the consequences of replication block due to the presence of secondary structures. A replication block at the secondary structure may cause disassembly of the replication complex, exposing the newly synthesized strand, which then becomes the recruiting center for the recombinational proteins. Alternatively, an endonucleolytic cleavage of the secondary structure can result in the formation of a DSB. In mitotic cells, the sister chromatids are preferentially used for recombination repair [52]. Accordingly, the presence of an IR increases the rate of spontaneous SCE; these SCE events occur by DSB repair [38]. In this study, we analyzed the effect of the *sgs1 *mutation and of mutations in genes that are functionally related to *SGS1 *on IR-associated spontaneous unequal SCE.

Sgs1 is functionally related to Srs2. Results from several genetic studies have suggested that Sgs1 and Srs2 deal with toxic recombination intermediates that arise during normal DNA replication by two separate mechanisms [24]. While Sgs1 resolves the toxic recombination intermediates, Srs2 limits the formation of such intermediates. A null mutation in either *SGS1 *or *SRS2 *is synthetically lethal with a mutation in several genes involved in DNA replication [25], and both *SGS1 *and *SRS2 *are implicated in the intra-S damage checkpoint mechanism [14,29]. The rates of spontaneous SCE events for both substrates in the *srs2 *background were increased. However, the fact that the rates were about 4–5 fold lower than those observed in the *sgs1 *background (Table 2) suggests that replication defects in *srs2 *cells are either repaired by Sgs1 or channeled into alternative repair pathways. For example, the otherwise toxic intermediates can be processed by the action of another helicase, such as Rrm3 [53,54].

Mus81 is believed to act in pathways parallel to those involving Sgs1 to resolve recombination intermediates generated during DNA replication [24]. The *mus81 *mutation exhibited a modest effect on SCE, suggesting that in *mus81 *cells, intermediates that are normally metabolized by Mus81 are either resolved by Sgs1 or else are repaired by pathways not involving SCE. It should be noted here that repair of a replication defect by SCE can occur by either equal or unequal SCE. Equal SCE is the predominant DSB repair mechanism [55]. In our system, we can detect only the unequal events. Therefore, we cannot rule out the possibility that the modest effect of the *srs2 *and *mus81 *mutations on unequal SCE is due to repair of replication defects by equal SCE.

The nature of the substrate that is metabolized by Sgs1 is not clear. During replication, DSBs are normally not generated in wild-type cells; recombination events are likely to be initiated on single-stranded gaps that form at stalled replication forks [24]. Accordingly, spontaneous SCE events are independent of genes involved in DSB repair; the rate of SCE remained close to the wild-type level in *rad51 *cells [38,56]. The IR-associated SCE events are, however, dependent on the DSB-repair enzymes, because IR-associated SCEs occur by DSB repair ([38]; Fig. 1). Since recombination enzymes function upstream of Sgs1, we expected spontaneous SCE to be reduced to the *rad51 *level, in the *sgs1 rad51 *double mutant. The spontaneous SCE rates in the *sgs1 rad51 *background for both substrates were reduced in the double mutant, but they nevertheless remained higher than the rate in the *rad51 *cells (Table 2). Similar results were also obtained by Spell and Jinks-Robertson [11], who found that homologous recombination in the *rad51 sgs1 *cells was reduced relative to the rate in *sgs1 *single mutant, although it remained higher than the wild-type level. These results suggest that both Rad51-dependent and Rad51-independent SCE events occur in the *sgs1 *background. In *S. cerevisiae*, some homologous recombination events are *RAD51*-independent but *RAD59*-dependent, but both types of event are *RAD52*-dependent [28], suggesting that Rad51-independent SCE events can occur by the Rad59 pathway.

The above conclusion, that various mechanisms operate in *SGS1 *cells to suppress spontaneous SCE, was supported by the result that 50% of the SCE events observed for *his3-SCS*_*pal*140 _in the *sgs1 *mutant are *MSH2*-dependent (Table 2). The role of Msh2 in generating IR-associated SCE events is not known. Myung *et al*. observed a reduction by slightly over 2-fold in the rate of homologous recombination with an IR substrate in the *sgs1 msh2 *double mutant [10]. In a separate study, Spell and Jinks-Robertson [11] found no difference in the rate of homologous recombination between the *sgs1 *single mutant and the *sgs1 msh2 *double mutant. However, neither of these results is directly comparable with our findings, because the substrate used by the two groups was not a perfect IR, but was interrupted by non-repeated sequences.

Only *MSH2 *and *MSH3 *of the MMR pathway are necessary for IR-associated SCE events [39]. IR-associated SCE events occur by DSB repair. If DSBs occur within the secondary structures, then the 3' end, after DSB formation and exonucleolytic processing, will contain non-homologous tails that must be removed for generating a wild-type *HIS3 *gene by unequal SCE. The Rad1/Rad10 endonuclease aided by the Msh2/Msh3 complex removes the non-homologous ends. The rate of SCE remains unaffected in the *rad1 *background (39), suggesting that non-homologous tails are removed by a mechanism that does not involve the Msh2/Msh3 complex. The Msh2/Msh3 complex is involved in loop repair [45]. The Msh2/Msh3 complex may recruit the processing enzyme to the stem-loop structure after DSB formation, or else Msh2/Msh3 could bind at the secondary structure, and then recruit the endonclease to generate the DSB.

These observations raise another issue: what enzyme is responsible for generating the break at the secondary structure? IR-associated SCE events are reduced in the *rad50 *and *mre11 *backgrounds [38]. Rad50 and Mre11 are components of the Mre11-Rad50-Xrs2 (MRX) complex that is required for DSB formation during meiosis, both at normal meiosis-specific sites and at IRs [28,57]. In mitotic cells, the MRX complex is required for DSB repair and for maintenance of the genome integrity [28]. Rad50 and Mre11 respectively show significant homology with the SbcC and SbcD proteins of *E. coli *[58]. The SbcCD complex is known to cleave hairpin structures [59]. Mre11 has been shown to cleave hairpin structures *in vitro *[60,61]. Therefore, one likely scenario is that the DSBs at the repeated sequences are generated via Mre11's hairpin cleavage activity. Our results on meiotic DSB formation in *mre11*-nuclease deficient cells (unpublished observation), and the results obtained by Resnick and his coworkers [62], suggest that Mre11's nuclease activity is not required for formation of DSBs during either meiosis or mitosis, but that it is necessary for processing of the DSBs at the secondary structure.

Exo1, like Sgs1, has been shown to act on the stalled replication fork [50]. The *exo1 *mutation is synthetically lethal with *rad27*, and overexpression of *EXO1 *suppresses several *rad27 *defects, suggesting that *EXO1 *is functionally redundant with *RAD27 *(*FEN1*) for Okazaki fragment processing [51]. However, the role of *EXO1 *in Okazaki fragment processing is unclear, because *exo1 *cells do not exhibit any growth defects or other phenotypes exhibited by the *rad27 *cells [51]. Perhaps, the observed increase in the rate of SCE in *exo1 *cells is due to inefficient processing of the stalled replication fork. The SCE rates in the *sgs1 exo1 *double mutant were synergistically increased for both substrates (Table 2), suggesting that *SGS1 *and *EXO1 *regulate spontaneous SCE by independent mechanisms. However, further studies are necessary to understand the underlying mechanisms of each of these two pathways. It is also not clear from the available data whether the increased level of SCE in *exo1 *cells occurs by the same mechanism for the two substrates. These events may occur by separate mechanisms, analogous to our results in *sgs1 *cells.

## Conclusions

IRs have the potential to form secondary structures, which are known to attenuate the normal progression of the replication fork. A block in the progression of the replication fork is likely to increase the rate of SCE. In this report, we studied the effects of mutations in *SGS1 *and in functionally related genes on IR-stimulated spontaneous unequal SCE. We conclude that 1) in wild-type cells, both IR and non-IR-associated spontaneous SCE events are suppressed by Sgs1; 2) the IR-associated SCE events in the *sgs1 *background are partially *MSH2*-dependent; 3) the increased level of SCE events in the *sgs1 *background arises via both *RAD51*-dependent and *RAD51*-independent pathways; however, most of the SCE events in the *sgs1 *background are *RAD52*-dependent; and 4) Sgs1 and Exo1 regulate spontaneous SCE events by independent mechanisms.

## Methods

### Yeast strains and plasmids

All yeast strains (Table 1) used in this study were derived from the AS13 (**a ***leu2-Bst ura3-52 ade6*) background [63]. DNY380 and DNY393 were constructed by introducing the control substrate and the IR-containing sister-chromatid recombination substrate, respectively, within the *ARG4 *locus. The construction of the control and the IR-containing SCSs, and of DNY380 and DNY393 has been described previously [38]. All genetic manipulations were carried out using standard procedures, and the media used are described by Rose *et al*. [64]. The *msh2*-mutant allele was introduced into the chromosome using the plasmid pII-2::Tn10LUK7-7, as described previously [39]. The *sgs1 *mutation was introduced using the plasmid pPWΔSGS1 [6], in which the *Hpa*I to *Eco*RV fragment was deleted from the *SGS1 *coding region and then replaced by the *LEU2 *gene. The plasmid was digested with *Nco*I and *Pst*I before transformation. The plasmid pPWΔSGS1 was kindly provided by Patrick Maxwell in Joan Curcio's laboratory (Wadsworth Center, Albany, NY). The *srs2::KANMX *allele was constructed with a PCR-generated fragment using primers 5' TTAAAACATGCTAGGGTAACGAGAC 3' and 5' ACTATTTTTGACTGGGTACTGCTTG 3' and DNA from the Resgene-deletion strain as a template. The *mus81::KAN *allele was generated using the oligonucleotides mus81-5', 5' ACCTATATATTGAATGGTTACAAGAATTAGTTGACGGATTG atcgatgaattcgagctcg 3' and mus81-3', 5' TCATATATCTTTTCTGAAAGAGATTTAGTAATTTTCTTCGTTcgtacgctgcaggtcgac 3', and pF6A [65] DNA as the template. The nucleotides in the lower case indicate sequences homologous to the KanMX cassette. The *exo1 *disruption was introduced into the chromosome using plasmid p245 as described previously [39]. The *rad51 *allele was introduced using *Bam*HI-digested pΔRAD51 [38]. The *rad52 *allele was introduced into the chromosome as described in ref. [38].

### Genetic analysis of unequal SCE

The rate of unequal SCE was determined by the method of median, as described previously [38]. Briefly, a single colony was inoculated into 3 ml of YPD broth and incubated at 30°C overnight. After suitable dilution, the culture was distributed into 13 tubes, each containing 3 ml of YPD broth. Each tube received about 10–20 cells. After 3–4 days of growth at 30°C, the cells were centrifuged, suspended in water, and sonicated briefly; they were then plated after suitable dilutions onto complete synthetic medium (CSM) to measure the total number of viable cells, and onto CSM lacking histidine (CSM-His) to determine the number of recombinants. Colonies were counted after 7 days of incubation at 30°C. For each strain, at least five independent rate calculations were performed using at least two different transformants, and the significance was determined by Students's *t*-test.

## List of Abbreviations

IR: inverted repeat; DSB: double-strand break; SCE: sister-chromatid exchange, CSM: complete synthetic medium; SCS: sister-chromatid recombination substrate.

## Authors' contributions

DKN designed the experiments, SJC and DKN performed the experiments and analyzed the data, DKN wrote the paper. SJC read and approved the final manuscript.
